# Supplementary material for: Extortion strategies resist disciplining when higher competitiveness is rewarded with extra gain
Source: Nat Commun. 2019 Feb 15;10:783. doi: 10.1038/s41467-019-08671-7 (PMC6377637; doi:10.1038/s41467-019-08671-7)
Supplement: Supplementary file 1 — Supplementary Information [file 41467_2019_8671_MOESM1_ESM.pdf]

Supplementary Information

Extortion strategies resist disciplining when higher competitiveness is rewarded with extra gain

Lutz Becks and Manfred Milinski

Supplementary Table 1. Statistical results for Fig. 1a. Random effect: pair nested within group. df_{nom}= degrees of freedom nominator, df_{denom}= degrees of freedom denominator. Player: potentially generous or partner

**Random effects**	**Variance**	**Std.Dev**		
Pair:group	0	0		
group	0	0		
Residual	0.001378	0.03712		
**Fixed effects**	**F**	**df_{nom}**	**df_{denom}**	**p-value**
cooperation rate	87.466	1	86	$9.326*10^{-15}$
player	15.624	1	86	0.000158
cooperation rate * player	6.418	1	96	0.013108

Supplementary Table 2. Statistical results for Fig. 1b. Random effect: pair nested within group. df_{nom}= degrees of freedom nominator, df_{denom}= degrees of freedom denominator. Player: potentially extortionate or partner

**Random effects**	**Variance**	**Std.Dev**		
Pair:group	$6.79*10^{-19}$	$8.24*10^{-10}$		
group	0	0		
Residual	$1.568*10^{-3}$	$3.96*10^{-2}$		
**Fixed effects**	**F**	**df_{nom}**	**df_{denom}**	**p-value**
cooperation rate	178.329	1	96	$< 2.2*10^{-16}$
player	1.185	1	96	0.279
cooperation rate * player	38.179	1	96	$1.557*10^{-8}$

Supplementary Table 3. Statistical results for Fig. 1c. Random effect: pair nested within group. df_{nom}= degrees of freedom nominator, df_{denom}= degrees of freedom denominator. Player: potentially extortionate or partner

**Random effects**	**Variance**	**Std.Dev**		
Pair:group	0	0		
group	0	0		
Residual	0.001436	0.0379		
**Fixed effects**	**F**	**df_{nom}**	**df_{denom}**	**p-value**
cooperation rate	178.329	1	96	$< 2.2*10^{-16}$
player	0.010	1	96	0.920087
cooperation rate * player	14.122	1	96	0.0002806

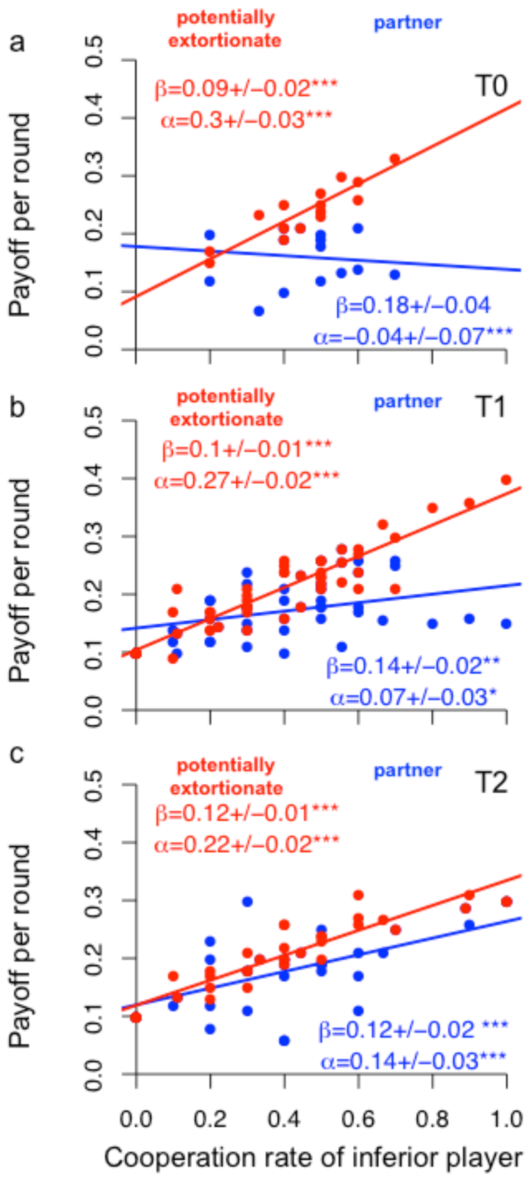

Supplementary Figure 1 Correlation between cooperation rate of the partner and payoff per round for the inferior player and partner. In the treatments **a** without incentive (T0), **b** with asymmetric incentive (T1), and **c** with symmetric incentive (T3). Individual points represent average payoff and the average cooperation of 10 rounds per individual. Slope and intercept

estimates (+/- s.e.m.) are derived from linear mixed effect models (for statistics see Supplementary Table 4). **a** potentially extortionate: R^2= 062; partner R^2= 0.91. **b** potentially extortionate: R^2=0.853; partner R^2= 0.40. **c** potentially extortionate: R^2= 0.85; partner R^2= 0.50. For all other players, see Fig. 2 in main text.

Supplementary Table 4. Statistical results for Supplementary Figure 1 a. Random effect: pair nested within group. df_{nom}= degrees of freedom nominator, df_{denom}= degrees of freedom denominator. Player: potentially extortionate or partner

**Random effects**	**Variance**	**Std.Dev**		
Pair:group	$3.036*10^{-5}$	0.005510		
group	$7.019*10^{-6}$	0.002649		
Residual	$1.392*10^{-3}$	0.037316		
**Fixed effects**	**F**	**df_{nom}**	**df_{denom}**	**p-value**
cooperation rate	10.0027	1	25	0.004060
player	1.199	2	24	0.284456
cooperation rate * player	8.184	2	24	0.008638

Supplementary Table 5. Statistical results for Supplementary Figure 1b. Random effect: pair nested within group. df_{nom}= degrees of freedom nominator, df_{denom}= degrees of freedom denominator. Player: potentially extortionate or partner

**Random effects**	**Variance**	**Std.Dev**		
Pair:group	0.0005344	0.02312		
group	0.0001100	0.01049		
Residual	0.0011873	0.03446		
**Fixed effects**	**F**	**df_{nom}**	**df_{denom}**	**p-value**
cooperation rate	63.647	1	61	$4.6 * 10^{-11}$
player	1.112	1	95	0.2942
cooperation rate * player	22.273	1	95	$8.136 * 10^{-6}$

Supplementary Table 6. Statistical results for Supplementary Figure 1c. Random effect: pair nested within group. df_{nom}= degrees of freedom nominator, df_{denom}= degrees of freedom denominator. Player: potentially extortionate or partner

**Random effects**	**Variance**	**Std.Dev**		
Pair:group	$8.184*10^{-5}$	0.009046		
group	0	0		
Residual	$1.585*10^{-3}$	0.039816		
**Fixed effects**	**F**	**df_{nom}**	**df_{denom}**	**p-value**
cooperation rate	53.204	1	38	$1.06*10^{-8}$
player	0.007	1	53	0.9318
cooperation rate * player	2.136	1	57	0.1493

Supplementary methods: Experimental game instructions.

In the beginning of our experiment, subjects were asked to read a few pages on their computer screens that would explain the rules of the subsequent game. In the following, we provide these instructions, translated from German.

Instructions for the treatment without incentive (T0):

Page 1.

Welcome to this experiment in which you can earn money. The exact amount

of money that you earn will depend on your own decisions and on the decisions of the other player. All decisions you make are anonymous. The computer program assigns you a pseudonym (Galatea, Nereid, Elara, Vestia, Leda and Setebos) and a randomly determined co-player.
At the end of the game, you will receive the money that you have earned in cash anonymously under your pseudonym.

Your co-player will remain the same for all round of the game.
In each round you and your co-player will have the same options to decide between.
A detailed explanation will follow on the next page.

After having read this text completely, please confirm by pressing the ‘Ok’-button.

Page 2:
In each decision situation, two players need to simultaneously choose a letter (either C or D). Each player needs to decide without knowing the choice of the co-player. Depending on your decision and on the decision of your co-player, you receive a certain payoff. The following table shows the possible payoffs. The first amount in each
Cell (green) corresponds to your own payoff, and the second amount (blue) corresponds to the payoff of your co-player.

		Decision of your co-player	
		C	D
Your	C	€0.30, €0.30	€0.00, €0.50
decision	D	€0.50, €0.00	€0.10, €0.10

After having read this text completely, please confirm by pressing the ‘Ok’-button.

Page 3:

Thus, there are four possible outcomes:

You: C Your co-player: C You get €0.30 Your co-player gets €0.30

You: C Your co-player: D You get €0.00 Your co-player gets €0.50

You: D Your co-player: C You get €0.50 Your co-player gets €0.00

You: D Your co-player: D You get €0.10 Your co-player gets €0.10

After having read this text completely, please confirm by pressing the ‘Ok’-button.

Page 4:

Examples: In each round you and your co-player will be asked: “Which letter do you want to choose (C or D)? After both of you have independently chosen a letter, the result will be shown to both of you.

Example 1: You have chosen C and you earn €0.30
Your co-player has chosen C and earns €0.30

Example 2: You have chosen D and you earn €0.50
Your co-player has chosen C and earns €0.00

Example 3: You have chosen D and you earn €0.10
Your co-player has chosen D and earns €0.10

Once both players have confirmed their outcome summary by clicking on the ‘Ok’ button, each player gets his respective amount credited to his account.

The experiment consists of many rounds. In all rounds you will have the same options to decide between (you can, however, in each round change your mind).

After having read this text completely, please confirm by pressing the ‘Ok’-button.

Page 5:

There is the possibility that you can receive a bonus after the game.

Bonus:

If both of you as a pair have earned on average at least €0.30 per round, both of you gets €5.00 credited to his account. You can achieve this only, if you as a pair make a certain proportion of C-C and/or C-D or D-C decisions. If both decide always D. you earn only €0.20 on average as a pair.

After having read this text completely, please confirm by pressing the ‘Ok’-button.

Now the rounds will start.

After having read this text completely, please confirm by pressing the ‘Ok’-button.

Page 6:

		Decision of your co-player	
		C	D
Your decision	C	€0.30, €0.30	€0.00, €0.50
	D	€0.50, €0.00	€0.10, €0.10

Your co-player is Nereid

In this round, do you want to decide C or D? …. C

…… D

Please confirm by pressing the ‘Ok’-button.

Page 7:

3. Commentary rounds

Now commentary rounds follow. You are asked to comment on your co-player’s strategy.
You are completely free how to comment.

However, you must not describe decisions with numbers (NOT:”3xC, 3xD”, “50% D”, 2xC” or similar.). The commentary must not be longer than 50 characters (= length of the available space for writing).

The commentary that you write about your co-player could potentially be the only information other players have about this player in further rounds. It may happen that your commentary could be the only decision-making help for a co-player in a pair round.

After having read this text completely, please confirm by pressing the ‘Ok’-button.

Page 8:

4. Evaluation rounds

Now we would like to ask you to assess all commentaries. These assessments should be honest and not strategic. (they will not be shown in the game).

You will see all commentaries sequentially. Please click on a number between 1 and 7, which is your assessment.

It is very important that the following is clear: 1 = positive, 4= neutral, 7= negative

After having read this text completely, please confirm by pressing the ‘Ok’-button.

Page 9:
Now you see the assessment of the decision behaviour of one player of the group: gossip about Vestia

How much would you like to be in a pair game with this player about whom this gossip had been written?

I would very much like to (1) ………….. not at all (7)

After having read this text completely, please confirm by pressing the ‘Ok’-button.

Page 10:
Now you see the assessment of the decision behaviour of one player of the group: gossip about Etebos

How much would you like to be in a pair game with this player about whom this gossip had been written?

I would very much like to (1) ………….. not at all (7)

After having read this text completely, please confirm by pressing the ‘Ok’-

button.

Page 11:

End of the game

Instructions with respect to gaining the extra bonus of €10 in T1:

Pages 1 to 4 are identical to those for T0.

Page 5:

There is the possibility that you can receive two kinds of bonus after the game.

Bonus 1:

This Bonus can be valid only for one of the two players. The computer will determine that person randomly. If this person has gained at least 10% more than his/her partner, he/she will receive €10 extra on his/her account.

Example:

If the randomly determined person has earned €10 at the end of the game and the partner has earned €9, the determined person has earned 10% more and will receive the extra bonus of €10.

Bonus 2:

If both of you as a pair have earned on average at least €0.30 per round, both of you gets €5.00 credited to his account. You can achieve this only, if you as a pair make a certain proportion of C-C and/or C-D or D-C decisions. If both decide always D. you earn only €0.20 on average as a pair.

After having read this text completely, please confirm by pressing the ‘Ok’-button.

Page 6:

The computer has randomly decided:

You will receive the extra bonus of €10, if you have earned after all rounds at least 10% more than your partner.

After having read this text completely, please confirm by pressing the ‘Ok’-button.

OR!!!

Page 6:

The computer has randomly decided:

Unfortunately you cannot win bonus1!

(Thus you will not receive the extra bonus of €10, if you have earned after all rounds at least 10% more than your partner)

After having read this text completely, please confirm by pressing the ‘Ok’-button.

Now the rounds will start.

After having read this text completely, please confirm by pressing the ‘Ok’-button.

Continued as in T0

Instructions with respect to gaining the extra bonus of €10 in T2:

Pages 1 to 4 are identical to those for T0.

Page 5:

There is the possibility that you can receive two kinds of bonus after the game.

Bonus 1:

This Bonus can be valid for each of the two players. However, only if one person has gained at least 10% more than his/her partner, he/she will receive €10 extra on his/her account. So only one player can win the bonus.

Example:

If one person has earned €10 at the end of the game and the partner has earned €9, the determined person has earned 10% more and will receive the extra bonus of €10.

Bonus 2:

If both of you as a pair have earned on average at least €0.30 per round, both of you gets €5.00 credited to his account. You can achieve this only, if you as a pair make a certain proportion of C-C and/or C-D or D-C decisions. If both decide always D. you earn only €0.20 on average as a pair.

After having read this text completely, please confirm by pressing the ‘Ok’-button.

Now the rounds will start.

After having read this text completely, please confirm by pressing the ‘Ok’-button.

Continued as in T0
