# Supplementary material for: Extortion strategies resist disciplining when higher competitiveness is rewarded with extra gain
Source: Nat Commun. 2019 Feb 15;10:783. doi: 10.1038/s41467-019-08671-7 (PMC6377637; doi:10.1038/s41467-019-08671-7)
Supplement: Supplementary file 3 — Reporting Summary [file 41467_2019_8671_MOESM3_ESM.pdf]

nature research

Corresponding author(s): Manfred Milinski

Reporting Summary

Nature Research wishes to improve the reproducibility of the work that we publish. This form provides structure for consistency and transparency in reporting. For further information on Nature Research policies, see Authors & Referees and the Editorial Policy Checklist.

Statistical parameters

When statistical analyses are reported, confirm that the following items are present in the relevant location (e.g. figure legend, table legend, main text, or Methods section).

n/a	Confirmed	
☐	☒	The exact sample size (*n*) for each experimental group/condition, given as a discrete number and unit of measurement
☐	☒	An indication of whether measurements were taken from distinct samples or whether the same sample was measured repeatedly
☐	☒	The statistical test(s) used AND whether they are one- or two-sided<br>*Only common tests should be described solely by name; describe more complex techniques in the Methods section.*
☐	☒	A description of all covariates tested
☐	☒	A description of any assumptions or corrections, such as tests of normality and adjustment for multiple comparisons
☐	☒	A full description of the statistics including central tendency (e.g. means) or other basic estimates (e.g. regression coefficient) AND variation (e.g. standard deviation) or associated estimates of uncertainty (e.g. confidence intervals)
☐	☒	For null hypothesis testing, the test statistic (e.g. *F*, *t*, *r*) with confidence intervals, effect sizes, degrees of freedom and *P* value noted<br>*Give P values as exact values whenever suitable.*
☒	☐	For Bayesian analysis, information on the choice of priors and Markov chain Monte Carlo settings
☒	☐	For hierarchical and complex designs, identification of the appropriate level for tests and full reporting of outcomes
☒	☐	Estimates of effect sizes (e.g. Cohen's *d*, Pearson's *r*), indicating how they were calculated
☐	☒	Clearly defined error bars<br>*State explicitly what error bars represent (e.g. SD, SE, CI)*

Our web collection on statistics for biologists may be useful.

Software and code

Policy information about availability of computer code

Data collection	Z-tree
Data analysis	R

For manuscripts utilizing custom algorithms or software that are central to the research but not yet described in published literature, software must be made available to editors/reviewers upon request. We strongly encourage code deposition in a community repository (e.g. GitHub). See the Nature Research guidelines for submitting code & software for further information.

Data

Policy information about availability of data

All manuscripts must include a data availability statement. This statement should provide the following information, where applicable:

- Accession codes, unique identifiers, or web links for publicly available datasets
- A list of figures that have associated raw data
- A description of any restrictions on data availability

all original data will be deposited at Dryad

Field-specific reporting

Please select the best fit for your research. If you are not sure, read the appropriate sections before making your selection.

☐ Life sciences ☒ Behavioural & social sciences ☐ Ecological, evolutionary & environmental sciences

For a reference copy of the document with all sections, see nature.com/authors/policies/ReportingSummary-flat.pdf

Behavioural & social sciences study design

All studies must disclose on these points even when the disclosure is negative.

Study description	Subjects participated in an iterated Prisoner's Dilemma game via an interactive computer Program (Z-tree)
Research sample	Kiel University under graduates, i.e. first semester biology students, age between 22 and 30 years, 60% females. Almost all students of the beginners course registered, the sample is thus representative for beginners of 2016. All groups were fluent in German language. We tried to find as many groups of 6 students as possible, knowing from experience from similar studies at least 10 statistical units (here PD groups) would be necessary per treatment.
Sampling strategy	All 3 PD groups of two subjects each of a session participated in the same treatment (the program could not handle different treatments simultaneously). The sequence of the 3 treatments was randomized for each experimental day. Samples sizes that could be achieved with that cohort were well above the size known from experience with similar experiments to be sufficient for statistical analysis.
Data collection	The experiment was performed via the Z-tree software from a server that was connected to individual laptops (on tables with a chair each) surrounded by opaque blinds separated by two seats from nearest neighbor. The researcher was in the same room behind additional blinds in case of failure of the program. The software collected all decisions of the participants under their individual pseudonyms, that had been randomly assigned to individual laptops. Participants had to draw lots to which laptop number they would be assigned in order to assure that they felt to be anonymous.
Timing	Data collection started on September 22, 2016 and ended on September 30, 2016.
Data exclusions	No data were excluded from the analyses, except for the 50th round that due to a program mistake was not recorded for all groups.
Non-participation	3 participants dropped out before the session started; they had health problems. They were replaced by students that were registered but could not be assigned to a session, because sessions were complete.
Randomization	Participants were randomly allocated to groups subject to availability of free places in sessions. 7 participants could not be allocated.

Reporting for specific materials, systems and methods

Materials & experimental systems

n/a	Involved in the study
☒	☐ Unique biological materials
☒	☐ Antibodies
☒	☐ Eukaryotic cell lines
☒	☐ Palaeontology
☒	☐ Animals and other organisms
☐	☒ Human research participants

Methods

n/a	Involved in the study
☒	☐ ChIP-seq
☒	☐ Flow cytometry
☒	☐ MRI-based neuroimaging

Human research participants

Policy information about studies involving human research participants

Population characteristics	First semester biology students of the University of Kiel, Germany, age between 22 and 30 years, 60 % females
Recruitment	Before a plenary lecture, each student received a flyer describing the procedure of planned experiment without telling what will be studied with an invitation to register on a specified web page, almost all students of that course participated
